# Key predictors of postpartum depression and anxiety symptoms among mothers in Kilifi, Kenya: a machine learning approach

**DOI:** 10.3389/fpsyt.2026.1790893

**Published:** 2026-03-17

**Authors:** Faith Neema Benson, Rachel Odhiambo, Willie Brink, Anthony K. Ngugi, Akbar K. Waljee, Eileen M. Weinheimer-Haus, Cheryl A. Moyer, Ji Zhu, Amina Abubakar

**Affiliations:** 1 Institute for Human Development, Aga Khan University, Nairobi, Kenya; 2 Department of Mathematical Sciences, Stellenbosch University, Stellenbosch, South Africa; 3 Department of Population Health, Medical College, Aga Khan University, Nairobi, Kenya; 4 Department of Learning Health Sciences, University of Michigan, Ann Arbor, MI, United States; 5 Center for Global Health Equity, University of Michigan, Ann Arbor, MI, United States; 6 Department of Statistics, University of Michigan, Ann Arbor, MI, United States; 7 Department of Psychiatry, University of Oxford, Oxford, United Kingdom; 8 Neuroscience Unit, Kenya Medical Research Institute (KEMRI)-Wellcome Trust Research Programme, Kilifi, Kenya

**Keywords:** machine learning, maternal mental health, postpartum anxiety, postpartum depression, predictive modeling

## Abstract

**Background:**

The burden of maternal postpartum depression and anxiety is disproportionately high in sub-Saharan Africa (SSA), yet the use of advanced analytical methods to capture the complex interplay of variables influencing these conditions remains underexplored.

**Objective:**

To apply machine learning (ML) methods to predict depressive and anxiety symptoms in postpartum mothers and to identify key and actionable predictors.

**Methods:**

This cross-sectional study included 1,995 biological mothers of singleton infants aged 0–6 months, using survey data collected between March 2023 and March 2024 in Kaloleni and Rabai sub-counties, Kilifi County, Kenya, within the Kaloleni–Rabai Health and Demographic Surveillance System. Depressive and anxiety symptoms were assessed using the Patient Health Questionnaire-9 and Generalized Anxiety Disorder-7, with scores ≥5 indicating symptoms. Potential features included sociodemographic, economic, nutritional, food insecurity, and health-related factors. Ridge Logistic Regression (LR), Random Forest (RF), and Extreme Gradient Boosting (XGBoost) models were applied to predict depressive and anxiety symptoms. Model performance was evaluated using area under the receiver operating characteristic curve (AUC), accuracy, sensitivity, and specificity. Shapley additive explanations values were used for feature selection and interpretation.

**Results:**

Among the 1,995 mothers, 15.1% had depressive symptoms, and 8.7% had anxiety symptoms. Model performance was acceptable and comparable across all models. For depression, AUC values for Ridge LR, RF and XGBoost were 0.724 (95% CI: 0.656–0.785), 0.711 (95% CI: 0.642–0.774), and 0.705 (95% CI: 0.628–0.772) respectively. For anxiety, AUCs were 0.788 (95% CI: 0.712–0.857), 0.789 (95% CI: 0.709–0.861), and 0.785 (95% CI: 0.708–0.854), respectively. Increased household food insecurity was the strongest predictor of both conditions. Additional key predictors included low wealth index, lower body mass index, higher number of children, pregnancy complications and advanced maternal age.

**Conclusions:**

Postpartum mental health disorders remain a substantial burden in SSA. This study demonstrates the feasibility of using ML to predict depressive and anxiety symptoms in postpartum mothers. The findings identify key predictors, notably increased household food insecurity, alongside socioeconomic status and maternal health characteristics, that could inform the design and testing of targeted interventions. Future studies should include external validation and examine causal links between these predictors and postpartum mental health outcomes.

## Introduction

Depression and anxiety are the most common maternal mental health disorders particularly during the postpartum period (1). Their burden is higher in low- and middle-income countries (LMICs), affecting 19%-22% of women for depression compared to 13%-15% in high-income countries (HICs) (2–4). Postpartum anxiety affects an estimated 15%-25% of women worldwide with higher rates in low-resource regions (4). In sub-Saharan Africa (SSA), prevalence is even higher, with depression ranging from 10%-70% and anxiety from 10%-50% (5–9). Postpartum depression and anxiety can impair daily functioning and, in severe cases, lead to suicidal thoughts (10). These disorders compromise maternal well-being and are linked to impaired child development, and reduced early childhood health (11–15). Identifying key predictors is therefore essential for designing targeted interventions and for future testing aimed at protecting both mothers and children.

Several studies have identified factors influencing depression in pregnant and postpartum women, including low education, young maternal age, smoking during pregnancy, poor economic status, marital issues, and lack of social support (14, 16–18). However, findings remain inconsistent. For example, Agnafors et al. found that younger maternal age was associated with maternal depression, while Muraca et al. identified advanced maternal age as a significant risk factor; both conducted in high income countries (HICs) (19, 20). In LMICs, income, socio-economic status and education have shown inconsistent associations with postnatal depression (21). Moreover, most of these studies used traditional statistical methods, such as inferential logistic regression, which are less suited for complex datasets, leading to misinterpretations (15, 17, 19, 20).

Advanced artificial intelligence (AI) techniques, specifically machine learning (ML) models, have shown promise in predicting mental health disorders such as depression, anxiety and post-traumatic stress disorder (22–26). These methods identify key predictors through importance rankings, inherently capturing nonlinear effects and complex interactions that conventional techniques often fail to address, thereby highlighting the most impactful points for development and future testing of targeted intervention. While some studies have applied ML methods to predict maternal mental health disorders, these have focused on cohorts from HICs (27–29), leaving a critical gap in the application and evaluation of such methods within LMICs.

This study applied ML methods to survey data in order to predict depressive and anxiety symptoms in postpartum mothers in rural Kenya. The study also identified key predictors with potential to inform the design and future testing of targeted interventions for maternal mental health and healthy child development in similar low-resource settings.

## Methods

### Study design, setting and participants

This cross-sectional study was conducted in Kaloleni and Rabai, two rural sub-counties in Kilifi County, Kenya, ranked among the poorest regions in the country (30, 31). The study analyzed baseline survey data from a prospective longitudinal study on early childhood development. Participants were identified through the Kaloleni-Rabai Health and Demographic Surveillance System, a population-based registry (32). Between March 2023 and March 2024, trained enumerators from the Institute for Human Development at Aga Khan University conducted household interviews using tablets equipped with the Open Data Kit. Data on sociodemographic, economic, nutritional, food insecurity, and health-related factors were collected as potential features, and anthropometric measurements were also obtained. Community Health Extension Workers and Community Health Promoters assisted in locating households. The study included 1,995 mothers of singleton infants aged 0–6 months who provided written informed consent and were assessed for depressive and anxiety symptoms. Eligible participants included mothers for whom the index infant was either their first child or a subsequent child, regardless of any previous successful or unsuccessful pregnancies. Mothers whose index infant was a multiple birth (e.g., twins or higher-order multiples) at the time of recruitment, as well as non-maternal caregivers, were excluded. This study adheres to the STROBE guidelines for cross-sectional studies (33). In addition, the TRIPOD+AI reporting guidelines (34) were followed.

### Variables

#### Outcomes

The main outcomes were depressive and anxiety symptoms, assessed using the Patient Health Questionnaire-9 (PHQ-9) (35) and the Generalized Anxiety Disorder-7 (GAD-7) (36) scales, respectively. The PHQ-9 scores range from 0 to 27, with scores of 5–9, 10–14, and 15–27 indicating mild, moderate, and severe depressive symptoms, respectively (35). Similarly, GAD-7 scores range from 0 to 21, with scores of 5–9, 10–14, and 15–21 representing mild, moderate, and severe anxiety symptoms, respectively (36). Both outcomes were dichotomized into binary variables, with scores ≥5 indicating the presence of depressive or anxiety symptoms (mild, moderate, or severe). This threshold is consistent with standard severity categories and was selected to maximize prevalence, similar to previous studies conducted in comparable settings (37–39). The internal consistency of the scales in this study was acceptable, with Cronbach’s alpha values of 0.84 (95% CI: 0.83–0.85) for PHQ-9 and 0.85 (95% CI: 0.84–0.86) for GAD-7. As a sensitivity analysis, we also evaluated a higher threshold of ≥10 for both depressive and anxiety symptoms. Threshold selection and sensitivity analysis were performed to assess the impact on prevalence, model stability, and predictive performance.

#### Potential features

The potential features included household food insecurity measured using the Household Food Insecurity Access Scale (40–43); sociodemographic characteristics such as age, education level, marital status, and socioeconomic status assessed using a 10-asset index commonly applied in the Kenyan context (44); health history information including pregnancy-related complications, delivery challenges, and other relevant health factors; and anthropometric measurements of height and weight, which were used to calculate body mass index (BMI) as an indicator of maternal nutritional status. A total of 37 potential features were selected based on prior literature and domain expertise. Detailed information on the measures used to collect these features is provided in **Supplementary Method 1**.

### Statistical analysis

#### Data preparation

Given the small number of missing values (**Supplementary Table 1**), imputation was performed using deterministic methods to ensure a complete and consistent dataset for model training. Numerical features were imputed using the median for its robustness to skewness and outliers, while categorical features were imputed using the mode. We acknowledge that deterministic imputation can underestimate variability and potentially introduce bias. However, given the very low proportion of missing data, any such bias is expected to be minimal. This approach allowed for a streamlined preprocessing workflow while preserving data integrity. Multiple imputation was not applied, as the limited missingness was unlikely to materially affect estimates and would have introduced additional methodological and computational complexity. Imputation was conducted prior to the training–testing split to preserve the full sample size and maintain consistent feature distributions. Ideally, imputation would be performed on the training data, with the resulting median and mode then applied to the test data. However, because the training–test split was random, any differences introduced by this approach are expected to be minimal. We also note that the outcome variable was not imputed. The maternal characteristics were summarized using descriptive statistics, with frequencies and proportions reported for categorical features and medians with interquartile ranges for continuous features (**Tables 1**, **2**). Data was processed and analyzed using Python software (version 3.11.1). Project materials, including code and documentation, are available at https://doi.org/10.17605/OSF.IO/4K6A7 (45).

**Table 1:** Participant characteristics (features) predicting depressive symptoms identified by ML models.

Characteristics (features)	Overall (N = 1995)	Postpartum depressive symptoms		P value
		No	Yes	
		(n=1693)	(n=302)(15.1%)	
Sociodemographic				
Age, years, median (IQR)	27.0 (23.0 to 32.0)	27.0 (23.0 to 32.0)	28.0 (24.0 to 33.0)	.006^b^
Number of pregnancies, median (IQR)	3.0 (2.0 to 5.0)	3.0 (1.0 to 5.0)	3.0 (2.0 to 5.0)	<.001^b^
Number of children, median (IQR)	3.0 (1.0 to 4.0)	3.0 (1.0 to 4.0)	3.0 (2.0 to 5.0)	<.001^b^
Number of children between 1 to 5 years, median (IQR)	1.0 (0.0 to 1.0)	1.0 (0.0 to 1.0)	1.0 (0.0 to 1.0)	.179^b^
Marital status, No. (%)				.001^a^
Married	1770 (88.7)	1520 (89.8)	250 (82.8)	
Unmarried	225 (11.3)	173 (10.2)	52 (17.2)	
Religion, No. (%)				.095^a^
Christian	1435 (71.9)	1217 (71.9)	218 (72.2)	
Islam	461 (23.1)	400 (23.6)	61 (20.2)	
Traditional	91 (4.6)	70 (4.1)	2 (0.7)	
Other	8 (0.4)	6 (0.4)	2 (0.7)	
Caring for children with disability, No. (%)				.018^c^
Yes	31 (1.6)	21 (1.2)	10 (3.3)	
No	1964 (98.4)	1672 (98.8)	292 (96.7)	
Socioeconomic				
Wealth index, median (IQR)	-0.2 (-0.3 to 0.1)	-0.2 (-0.3 to 0.1)	-0.2 (-0.3 to 0.1)	.874^b^
Health history				
Pregnancy complications, No. (%)				<.001^a^
Yes	218 (10.9)	157 (9.3)	61 (20.2)	
No	1777 (89.1)	1536 (90.7)	241 (79.8)	
Child survival in all pregnancies, No. (%)				.018^a^
Yes	1663 (83.4)	1424 (84.1)	239 (79.1)	
No	332 (16.6)	269 (15.9)	63 (20.9)	
Household food insecurity scores, median (IQR)	7.0 (1.0 to 13.0)	6.0 (0.0 to 11.0)	13.0 (8.0 to 17.0)	<.001^b^
Nutrition				
Maternal BMI, median (IQR)	21.2 (19.3 to 24.0)	21.2 (19.4 to 24.0)	20.9 (18.7 to 23.7)	.056^b^

^a^ refers to Pearson’s χ².

^b^ refers to the Kruskal-Wallis test.

^c^ refers to Fisher’s exact tests.

**Table 2:** Participant characteristics (features) predicting anxiety symptoms identified by ML models.

Characteristics (features)	Overall N=1995	Postpartum anxiety symptoms		P value
	(N = 1995)	No	Yes	
		(n=1822)	(n=173) (8.7%)	
Sociodemographic				
Age, years, median (IQR)	27.0 (23.0 to 32.0)	27.0 (23.0 to 32.0)	28.0 (24.0 to 34.0)	.004^b^
Number of pregnant, median (IQR)	3.0 (2.0 to 5.0)	3.0 (1.0 to 5.0)	3.0 (2.0 to 5.0)	.007^b^
Number of children, median (IQR)	3.0 (1.0 to 4.0)	3.0 (1.0 to 4.0)	3.0 (2.0 to 5.0)	.008^b^
Number of children between 1 and 5, years, median (IQR)	1.0 (0.0 to 1.0)	1.0 (0.0 to 1.0)	1.0 (0.0 to 1.0)	.365^b^
Religion, No. (%)				.391^a^
Christian	1435 (71.9)	1302 (71.5)	133 (76.9)	
Islam	461 (23.1)	430 (23.6)	31 (17.9)	
Traditional	91 (4.6)	83 (4.6)	8 (4.6)	
Other	8 (0.4)	7 (0.4)	1 (0.6)	
Caring for children with disability, No. (%)				.183^c^
Yes	31 (1.6)	26 (1.4)	5 (2.9)	
No	1964 (98.4)	1796 (98.6)	168 (97.1)	
Socioeconomic				
Wealth index, median (IQR)	-0.2 (-0.3 to 0.1)	-0.2 (-0.3 to 0.1)	-0.1 (-0.3 to 0.2)	.598^b^
Education status, No. (%)				.826^a^
None (No formal education)	317 (15.9)	288 (15.8)	29 (16.8)	
Formal education	1678 (84.1)	1534 (84.2)	144 (83.2)	
Health history				
Pregnancy complication, No. (%)				<.001^a^
Yes	218 (10.9)	182 (10.0)	36 (20.8)	
No	1777 (89.1)	1640 (90.0)	137 (79.2)	
Place of delivery, No. (%)				.508^a^
Home	218 (10.9)	196 (10.8)	22 (12.7)	
Hospital/clinic	1777 (89.1	1626 (89.2)	151 (87.3)	
Child survival in all pregnancies, No. (%)				.012^a^
Yes	1663 (83.4)	1531 (84.0)	132 (76.3)	
No	332 (16.6)	291 (16.0)	41 (23.7)	
Attended antenatal clinic, No. (%)				.017^a^
Yes	1901 (95.3)	1743 (95.7)	158 (91.3)	
No	94 (4.7)	79 (4.3)	15 (8.7)	
Household food insecurity scores, median (IQR)	7.0 (1.0 to 13.0)	6.0 (1.0 to 12.0)	13.0 (8.0 to 17.0)	<.001^b^
Nutrition				
Maternal BMI, median (IQR)	21.2 (19.3 to 24.0)	21.2 (19.4 to 24.0)	21.1 (18.9 to 24.1)	.382^b^

^a^ refers to Pearson’s χ².

^b^ refers to the Kruskal-Wallis test.

^c^ refers to Fisher’s exact tests.

#### Exploratory data analysis

Group differences in participant characteristics were compared using Pearson’s χ² and Fisher’s exact tests for categorical data, and the Kruskal-Wallis test for data with a non-normal distribution. The TableOne package in Python was used, with the ‘*htest_name=True’* option enabled to display the names of the statistical tests applied. Fisher’s exact test was automatically used for categorical features when appropriate, while Pearson’s χ² test was applied otherwise. Hypothesis tests were conducted with a two-tailed approach, and statistical significance was set at *P* <.05. For continuous variables, Pearson correlation coefficients were computed and plotted in a pairwise manner for all features identified as important or tentative. Decisions on feature retention were guided by expert input, the impact on predictive performance, and the ability of the selected models to handle multicollinearity, as discussed below. Similarly, for categorical features, Cramér’s V correlation coefficients were calculated and plotted pairwise, and feature retention was guided by expert knowledge. Categorical features were encoded using the one-hot encoding method to allow easier interpretability of model outputs (**Supplementary Table 2**).

#### Model development

This study investigated three supervised ML models (classifiers): a traditional Ridge Logistic Regression (LR) and the more advanced Random Forest (RF) and Extreme Gradient Boosting (XGBoost). These algorithms were chosen for their efficiency, widespread application in healthcare research, and their potential for explainability (46, 47). Ridge LR was considered, rather than standard logistic regression, to address potential multicollinearity, as it penalizes large coefficients and stabilizes estimates when features are correlated (48–50). RF and XGBoost are relatively insensitive to multicollinearity because, as tree-based models, they make splits based on decision rules, typically selecting one of the correlated features at each split, with highly correlated features being selected at different splits, and thereby preserving predictive performance (51, 52). The models were implemented using the *sklearn.linear_model* library for LR, *sklearn.ensemble* for RF, and the *xgboost* library for XGBoost.

The model development process began with stratifying the dataset by the outcome variable to ensure balanced representation in both the training and test sets. Following the commonly used 80:20 data splitting ratio (53), the dataset (n=1,995) was randomly divided into 80% training and 20% testing for both outcomes (anxiety and depressive symptoms). This sample size provided sufficient data to train and evaluate the ML models, falling within published ranges for Ridge LR, RF, and XGBoost sample size requirements (54). It is also consistent with traditional events-per-variable (EPV) recommendations, which suggest at least 10 events per variable (feature) for reliable estimation (55). For depressive symptoms, the EPV is approximately 13, exceeding this guideline, while for anxiety symptoms the EPV is about 7.6, slightly below the guideline. Given the moderate number of features and the use of regularized models (e.g., Ridge LR, RF, and XGBoost), stable estimation and minimal overfitting are expected for both outcomes.

To address the issue of class imbalance efficiently, class weights were adjusted using the *class_weight=‘balanced’* parameter in the RF and LR algorithms (56). This method automatically assigns weights inversely proportional to class frequencies in the input data, promoting fairness in the model’s predictions. In contrast, for the XGBoost model, the *scale_pos_weight* parameter was utilized, calculated as the ratio of the positive class proportion to the negative class proportion (57). This adjustment ensures that the model gives appropriate emphasis to the minority class, enhancing its performance in imbalanced datasets.

To fine-tune the models and select the best hyperparameters (**Supplementary Table 3**), 10-fold cross-validation was applied to the training set to reduce the risk of overfitting. This involved dividing the training data into 10 equal parts. At each fold, 90% of the training data was used for model training, while the remaining 10% was used for validation (58).

Grid search was used for hyperparameter tuning to identify the optimal model, with performance evaluated through 10-fold cross-validation as mentioned above and the area under the receiver operating characteristic curve (AUC). This metric provided insights into each classifier’s ability to distinguish between positive and negative classes, particularly in scenarios of class imbalance (59).

#### Feature selection

The feature selection process was based on feature importance rankings derived from SHapley Additive exPlanations (SHAP) values, as proposed by Lundberg et al. (60) The SHAP offers a more consistent method for calculating feature importance and effects compared to traditional methods like gain-based importance in ensemble tree models. Drawing on game theory, SHAP fairly distributes the value among features based on their contribution to the model’s predictions. SHAP version 0.44.1 was used.

#### Model performance evaluation

After obtaining the optimal model, its performance was evaluated using the remaining 20% of the dataset for both outcomes, which had been reserved for testing. These test sets provided an evaluation of how well the model would generalize to unseen data, as they had not been utilized in the training or validation phases. Various performance metrics, including accuracy, sensitivity, and specificity, were employed to gain a comprehensive understanding of the model’s performance (61). The Youden Index was employed to identify the optimal classification cutoff (62). However, this cutoff can be adjusted depending on which metric (sensitivity, specificity, or overall accuracy) is prioritized for a particular clinical or research goal. Additionally, the AUC was calculated to assess the model’s ability to discriminate between different outcome classes across various decision thresholds (63). Bootstrapping with 1,000 resamples was used to generate confidence intervals for performance metrics on the test data. This internal resampling validation reduced variability due to sample composition and ensured more reliable inference. Since the test data were drawn from the same original dataset, this evaluation represents an internal validation. The calibration of the models was evaluated using the Hosmer–Lemeshow goodness of fit test, Brier score, and the model’s calibration curve.

#### Model interpretation/explainability

The explainability of the best-performing models on internal validation was explored by examining feature importance and feature impact using SHAP values. Two SHAP methods were used: SHAP dependence plots, which visualize the relationship between individual features and the model’s predictions, and SHAP summary plots (bar plots and beeswarm plots), which display feature importance and direction of impact, highlighting the key predictors. All SHAP plots were generated using the test data to assess feature importance and impact on the prediction.

## Results

The characteristics of the 1,995 mothers (median age [IQR], 27 [23–32] years) of singleton infants aged 0–6 months are presented in **Tables 1** and **2**. Based on a cutoff score of ≥5 (mild to severe), 302 mothers (15.1%) exhibited depressive symptoms and 173 (8.7%) exhibited anxiety symptoms. A detailed description of participants across all 37 features considered as potential features is provided in **Supplementary Table 1**.

Significant group differences were observed in most of the characteristics for both outcomes (P <.05, **Tables 1**, **2**). The correlation matrices for both numerical and categorical features are presented in **Supplementary Figures 1**, **2**. Among the numerical features, the number of children and the number of pregnancies were highly correlated (r = 0.96). Both were retained in the modeling process based on domain expert advice, as they capture distinct aspects of reproductive history: the number of children represents the total number of living children, while the number of pregnancies encompasses the full pregnancy history, including live births and miscarriages. Similarly, although living with a partner and marital status showed a strong correlation (r = 0.87), and place of delivery and delivery assistant were also highly correlated (r = 0.97), both pairs were retained based on expert guidance. Importantly, the correlated features were retained because the analysis employed methods capable of handling multicollinearity, including Ridge LR, RF, and XGBoost.

### Model performance evaluation

**Figure 1:**
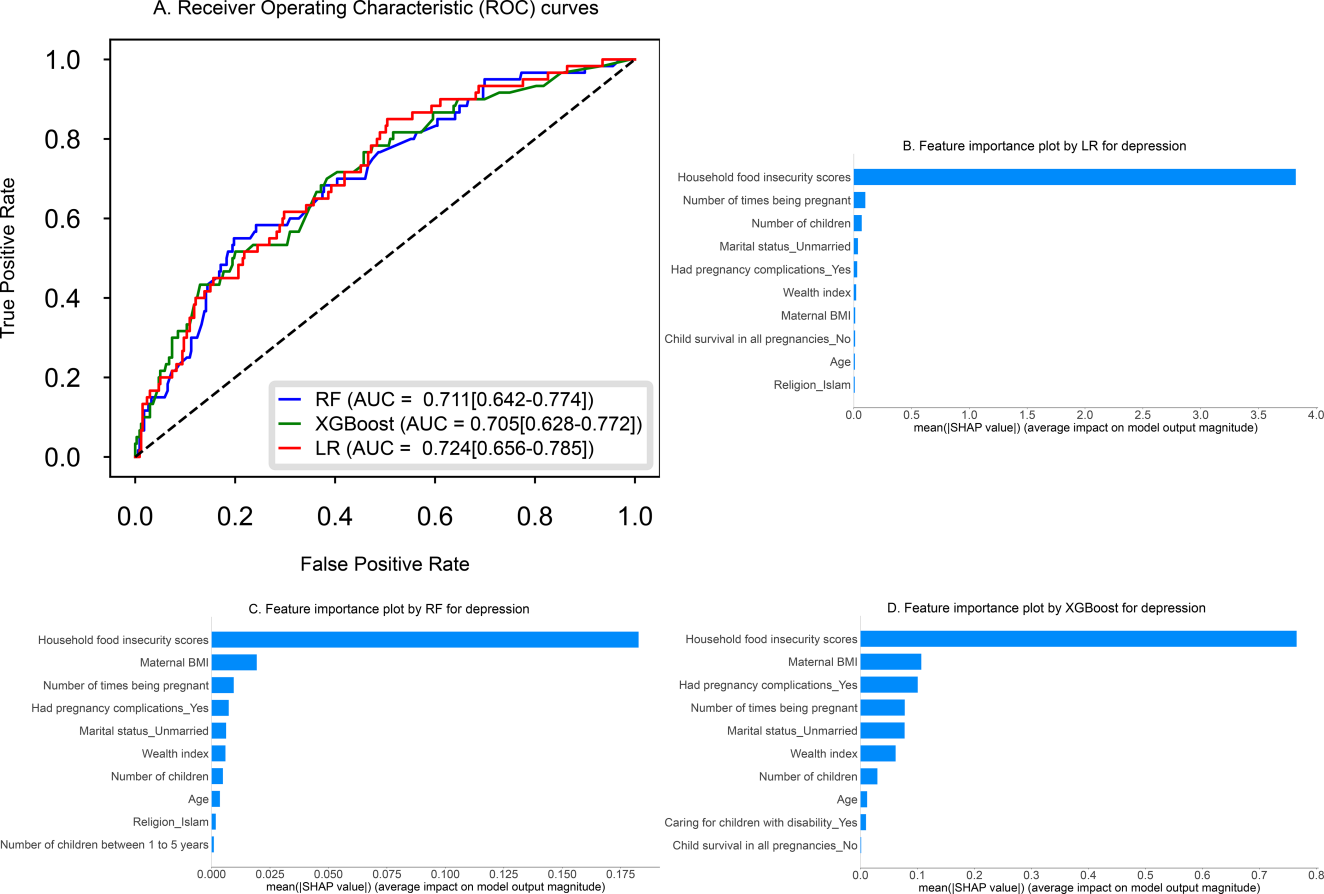
Plot **(A)** presents the Receiver Operating Characteristic (ROC) curves for three models, Ridge Logistic Regression (LR), Random Forest (RF), and Extreme Gradient Boosting (XGBoost), along with their corresponding AUC values for predicting depressive symptoms. SHAP feature importance plots **(B–D)** display the top 10 important features influencing depressive symptoms, ranked from most to least important.

**Figure 2:**
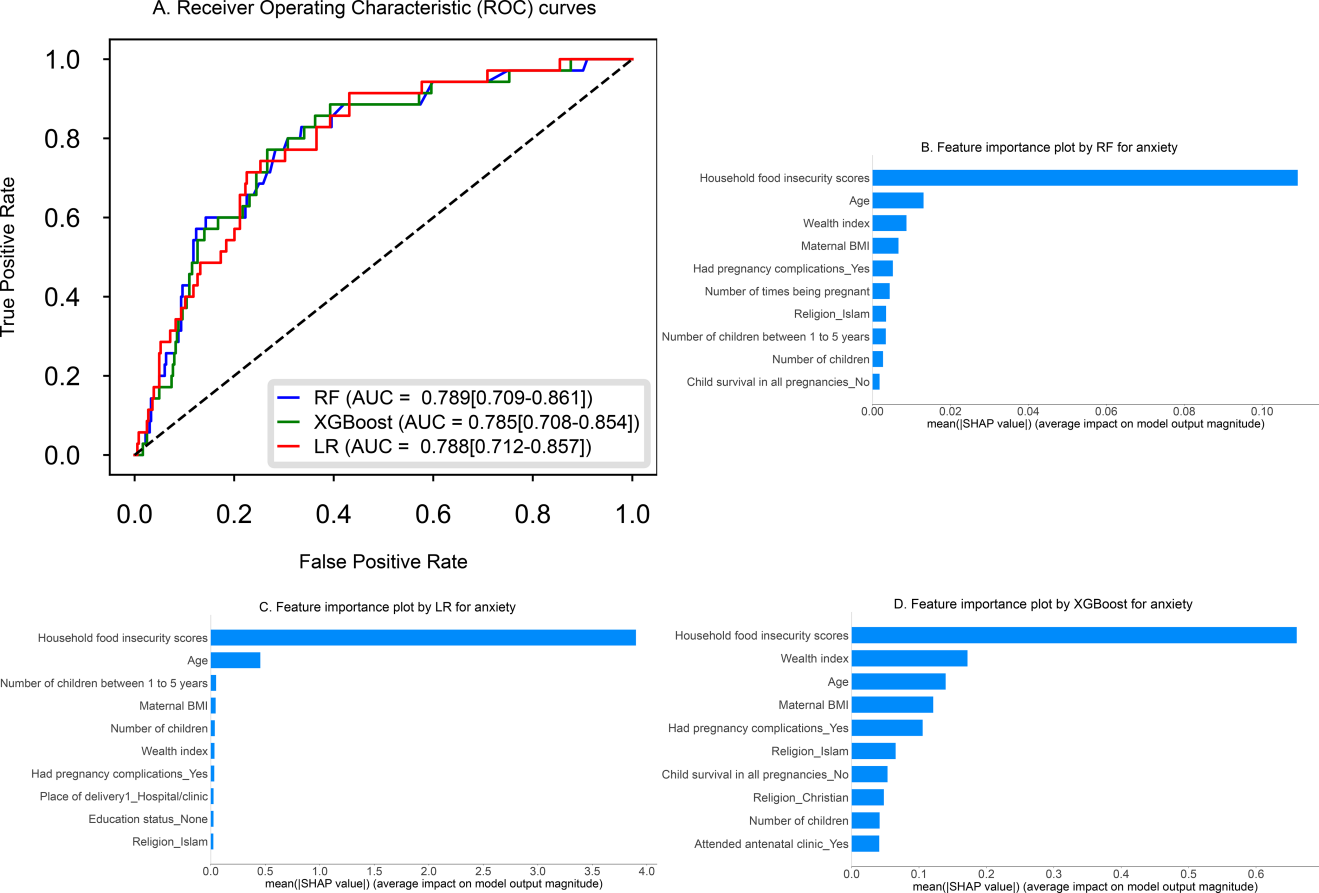
Plot **(A)** presents the Receiver Operating Characteristic (ROC) curves for three models, Ridge Logistic Regression (LR), Random Forest (RF), and Extreme Gradient Boosting (XGBoost), along with their corresponding AUC values for predicting anxiety symptoms. SHAP feature importance plots **(B–D)** display the top 10 important features influencing anxiety symptoms, ranked from most to least important.

**Table 3** summarizes the performance of all three models for depression and anxiety symptoms, each achieving AUCs above 0.70 on unseen test data using 23 features. **Figures 1A**, **2A** present the receiver operating characteristic curves for predicting both outcomes. In predicting depressive symptoms, Ridge LR, RF and XGBoost demonstrated similar performance, with AUCs of 0.724 (95% CI: 0.656–0.785), 0.711 (95% CI: 0.642–0.774) and 0.705 (95% CI: 0.628–0.772), respectively. Detailed performance metrics such as accuracy, sensitivity, and specificity are also included in **Table 3**. On the test set, the Brier scores were 0.117, 0.119, and 0.118 for Ridge LR, RF, and XGBoost, respectively. The Hosmer–Lemeshow goodness-of-fit test indicated no evidence of poor calibration for any model (*P* > 0.05; *P* = 0.203 for Ridge LR, *P* = 0.254 for RF, and *P* = 0.595 for XGBoost). Both the calibration curves (**Supplementary Figure 6A**) and Hosmer–Lemeshow test indicate that the predicted probabilities closely match the observed outcomes, demonstrating that the models are well-calibrated on the test set. When limited to the top 10 SHAP-selected features, the models maintained comparable performance, with AUCs of 0.723 (95% CI: 0.652–0.783) for Ridge LR, 0.708 (95% CI: 0.638–0.772) for RF and 0.705 (95% CI: 0.630–0.772) for XGBoost.

**Table 3:** Evaluation of model performance on unseen test data.

Outcome variable	Model	No of features	Test size	Best cutoff	Sensitivity (95%CI)	Specificity (95%CI)	AUC (95%CI)	Accuracy (95%CI)
Depression	Ridge Logistic regression	23	399 (20%)	0.486	0.633 [0.629 - 0.637]	0.711 [0.709 - 0.713]	0.724 [0.656 - 0.785]	0.699 [0.698 - 0.701]
		10	399 (20%)	0.480	0.617 [0.613 - 0.621]	0.693 [0.692 - 0.695]	0.723 [0.652 - 0.783]	0.682 [0.680 - 0.683]
	XGBoost	23	399 (20%)	0.536	0.533 [0.529 - 0.537]	0.717 [0.715 - 0.718]	0.705 [0.628 - 0.772]	0.689 [0.688 - 0.691]
		10	399 (20%)	0.538	0.533 [0.529 - 0.537]	0.717 [0.715 - 0.718]	0.705 [0.630 - 0.772]	0.689 [0.688 - 0.691]
	Random forest	23	399 (20%)	0.558	0.583 [0.579 - 0.587]	0.714 [0.712 - 0.715]	0.711 [0.642 - 0.774]	0.694 [0.693 - 0.696]
		10	399 (20%)	0.551	0.583 [0.579 - 0.587]	0.723 [0.721 - 0.724]	0.708 [0.638 - 0.772]	0.702 [0.700 - 0.703]
Anxiety	Ridge Logistic regression	23	399 (20%)	0.497	0.743 [0.738 - 0.748]	0.701 [0.699 - 0.702]	0.788 [0.712 - 0.857]	0.704 [0.703 - 0.706]
		10	399 (20%)	0.490	0.714 [0.710- 0.719]	0.684 [0.683 - 0.686]	0.786 [0.708 - 0.859]	0.687 [0.685 - 0.688]
	XGBoost	23	399 (20%)	0.520	0.657 [0.652 - 0.662]	0.739 [0.738 - 0.741]	0.785 [0.708 - 0.854]	0.732 [0.730 - 0.733]
		10	399 (20%)	0.529	0.714 [0.710 - 0.719]	0.736 [0.735 - 0.738]	0.792 [0.709 - 0.867]	0.734 [0.733 - 0.736]
	Random forest	23	399 (20%)	0.491	0.743 [0.738 - 0.748]	0.728 [0.727 - 0.730]	0.789 [0.709 - 0.861]	0.729 [0.728- 0.731]
		10	399 (20%)	0.466	0.686 [0.681 - 0.691]	0.739 [0.738 - 0.741]	0.784 [0.701, 0.855]	0.734 [0.733 - 0.736]

In predicting anxiety symptoms, Ridge LR, RF and XGBoost performed similarly, with AUCs of 0.788 (95% CI: 0.712–0.857), 0.789 (95% CI: 0.709–0.861) and 0.785 (95% CI: 0.708–0.854), respectively. Additional performance metrics, including accuracy, sensitivity, and specificity, are also presented in **Table 3**. On the test set, the Brier scores were identical across models (0.074 for Ridge LR, RF, and XGBoost). The Hosmer–Lemeshow goodness-of-fit test showed no evidence of poor calibration (*P* > 0.05; *P* = 0.069 for Ridge LR, *P* = 0.494 for RF, and *P* = 0.066 for XGBoost). Both the calibration curves (**Supplementary Figure 6B**) and Hosmer–Lemeshow test indicate that the predicted probabilities closely match the observed outcomes, demonstrating that the models are well-calibrated on the test set. When using a reduced set of the 10 most important features selected via the SHAP method, model performance remained stable. Ridge LR achieved an AUC of 0.786 (95% CI: 0.708–0.859), with RF at 0.784 (95% CI: 0.701–0.855) and XGBoost at 0.792 (95% CI: 0.709–0.867).

### Model interpretation/explainability

**Figures 1B–D**, **2B–D** show that the top 10 important features included household food insecurity, wealth index, maternal age, maternal BMI, pregnancy complications and number of children. These appeared consistently across all models and the remaining important features are shown in the figures. **Supplementary Figures 3A**, **4A** illustrate the magnitude and direction of impact (positive or negative) of these features on the prediction of depressive and anxiety symptoms, highlighting the key predictors.

**Supplementary Figures 3B–G**, **4B–G** present partial dependence plots for the top 6 key predictors generated by the RF model, selected for its interpretability of nonlinear feature–outcome relationships. Increased food insecurity, higher number of pregnancies, and low maternal BMI had a positive impact on predicting depressive and anxiety symptoms. In addition, low wealth index, pregnancy complications, and being unmarried had a positive impact on predicting depressive and anxiety symptoms.

### Sensitivity analysis

Applying the conventional ≥10 threshold for depressive symptoms substantially reduced the number of positive cases, resulting in a prevalence of 2% and pronounced class imbalance. This adversely affected model stability and predictive performance. For anxiety, the prevalence was even lower (0.6%), and test sets generated through random splitting often contained no positive cases, limiting the reliability of discrimination metrics. For depressive symptoms, the low number of cases resulted in wide confidence intervals around performance estimates (AUC 0.83, 95% CI 0.65–0.98), reflecting uncertainty due to the limited sample size. These results highlight the challenges of using higher thresholds in datasets with low prevalence.

## Discussion

To our knowledge, this is the first study to apply ML methods to survey data in order to predict depressive and anxiety symptoms in postpartum mothers in Kenya. We found that the RF and XGBoost models performed similarly to the traditional Ridge LR model in predicting both depressive and anxiety symptoms. Interestingly, SHAP analysis identified increased household food insecurity as the strongest predictor associated with model predictions for both conditions. These findings underscore the substantial burden of postpartum mental health disorders in Kenya and highlight increased household food insecurity as a key predictor that may inform the prioritization of factors for future intervention research.

The prevalence of postpartum depressive (15.1%) and anxiety (8.7%) symptoms in our study aligns with previous findings from Kenya, where studies have reported prevalences of postpartum depressive symptoms ranging from 13%-17% (64–67) and one study documented an anxiety symptom prevalence of 6.4% (68). These estimates are also consistent with reports from other LMICs, where postpartum depression ranges from 10%-25% and anxiety from 8%-16% (69, 70). Variations across studies may reflect differences in screening tools, population characteristics, and study designs. Importantly, we defined depression and anxiety symptoms using PHQ-9 and GAD-7 scores of ≥5, respectively, which is consistent with established severity categorizations in screening practice (scores 5–9: mild symptoms; ≥10: moderate to severe symptoms). The ≥5 cutoff is particularly relevant for community and primary care settings, where early identification of depressive and anxiety symptoms can prompt timely follow-up, further evaluation, or supportive interventions. While a cutoff of ≥10 is more specific for probable major depressive disorder and may be more appropriate for diagnostic or treatment pathways, the lower threshold supports preventive and public health–oriented approaches by identifying individuals who may benefit from psychosocial support or monitoring before symptom escalation.

Our findings demonstrate that, even in a low-resource context, both advanced ML (RF, XGBoost) and traditional (Ridge LR) models can identify maternal mental health predictors with potential relevance for targeted interventions. All three models demonstrated acceptable discriminative ability (AUC > 0.70) in identifying postpartum mothers with depressive or anxiety symptoms using only ten selected features (71). Prior studies from HICs have found similar results using data from electronic health records (EHRs) (27–29). In the current study, we utilized survey data which is likely more accessible in LMICs where EHRs are less common, especially in rural areas. In the context of LMICs, the comparable performance of Ridge LR to more complex models highlights its practicality for maternal mental health screening with modest datasets, given its interpretability, low resource requirements, and ease of implementation, whereas more complex models may offer improved performance with larger datasets (72).

Key predictors of postpartum depressive and anxiety symptoms identified using SHAP analysis included increased household food insecurity, low wealth index, low maternal BMI, higher number of children, pregnancy complications, and advanced maternal age. These predictors consistently emerged across all three evaluated models and were strongly associated with higher predicted probabilities of both depressive and anxiety symptoms. These findings align with prior evidence using traditional statistical methods, which also show that socioeconomic and health-related vulnerabilities drive maternal mental health (19, 73–76). However, some contrasts emerged with studies using traditional methods, such as reports of higher risk among younger mothers or links between obesity and greater depressive and anxiety symptoms (20, 77), which may reflect contextual differences or the limitations of traditional approaches in handling complex datasets. Nonetheless, increased household food insecurity consistently emerged as the strongest predictor of both depressive and anxiety symptoms in this study, highlighting its priority when selecting factors to consider in the design and evaluation of future interventions. It is important to note that SHAP values reflect the contribution of each feature to the model’s predictions and do not imply causal relationships. If causal relationships are confirmed in future studies, integrated approaches such as the Maternal, Infant, and Young Child Nutrition support groups initiated by the USAID Stawisha Pwani project in Kwale County, which combine health education with income-generating activities including beekeeping, kitchen gardening, and small animal rearing (such as rabbits and poultry), with surplus produce sold at local markets, could represent a promising strategy to address this challenge (77).

Additional predictors included lack of formal education, number of pregnancies, marital status, antenatal clinic attendance, place of delivery, child survival, caring for children with disabilities, and religion. These appeared less consistently, suggesting more context-specific effects. Religion, in particular, interacted with socioeconomic factors (food insecurity, wealth, BMI), with stronger effects observed among Christians than Muslims (**Supplementary Figure 5**). These interaction patterns may reflect differential social or contextual influences across religious groups; however, as they were identified through exploratory analyses, they should be interpreted cautiously and imply neither causal nor confounding effects.

### Strengths and limitations

This study employed robust methods, including validated screening tools (PHQ-9, GAD-7) and ML models for predictive modeling. A key strength of this study is the use of established reporting guidelines, with adherence to STROBE for cross-sectional studies and TRIPOD+AI for transparent reporting of ML–based prediction models. The use of SHAP values enhanced interpretability, offering actionable insights for policymakers and practitioners. Model calibration and goodness-of-fit assessments demonstrated that predicted probabilities closely matched observed outcomes, highlighting the reliability of the models.

This study has several limitations. The moderate sample size and imbalanced dataset may have restricted predictive power, and data collection via interviews could have introduced bias, particularly underreporting of symptoms due to stigma. Key psychosocial predictors, such as social support, intimate partner violence, and partner mental health, were not captured, limiting the scope of the models. Additionally, these findings are based on data from Kilifi County, which has unique characteristics and may not be generalizable to other settings in Kenya or SSA; further external validation is needed to assess applicability in similar contexts. As this cross-sectional study focused on mothers of infants aged 0–6 months, ongoing longitudinal studies will be valuable for assessing temporal stability and strengthening the robustness of the findings. Lastly, SHAP values are designed to explain model behavior rather than establish causality. Accordingly, SHAP-based interpretations should be considered associative and hypothesis-generating. Future research is needed to determine whether the identified predictors have a causal impact on postpartum mental health outcomes.

## Conclusion

To date, this is the first study to apply ML methods to survey data to predict depressive and anxiety symptoms in postpartum mothers in Kenya. These findings underscore the burden of mental health disorders in postpartum mothers in rural Kenya and demonstrate the feasibility of using ML to predict depressive and anxiety symptoms in postpartum mothers; however, further external validation is needed. Increased household food insecurity emerged as the strongest predictor for both conditions. Additional key predictors included low wealth index, low maternal BMI, higher number of children, pregnancy complications, and advanced maternal age. The identified predictors have the potential to inform the design and future evaluation of interventions. Future research could explore causal relationships between these predictors and postpartum mental health outcomes.

## Data Availability

The raw data supporting the conclusions of this article will be made available by the authors, without undue reservation.
