# Supplementary material for: Key predictors of postpartum depression and anxiety symptoms among mothers in Kilifi, Kenya: a machine learning approach
Source: Front Psychiatry. 2026 Mar 17;17:1790893. doi: 10.3389/fpsyt.2026.1790893 (PMC13036147; doi:10.3389/fpsyt.2026.1790893)
Supplement: Supplementary file 1 [file SupplementaryFile1.pdf]

Table of contents

Supplementary Table 1. Potential features and their level of missingness................................ 2

Supplementary Table 2. Features retained after one hot encoding... 3

Supplementary Table 3. Hyperparameter tuning, optimal values for each model and random seed... 3

Supplementary Figure 1. Correlation matrix of numerical features.. 4

Supplementary Figure 2. Correlation matrix of categorical features 5

Supplementary Figure 3. SHAP impact and partial dependence plots for predicting depressive symptoms by Random Forest 6

Supplementary Figure 4. SHAP impact and partial dependence plots for predicting anxiety symptoms by Random Forest .... **Error! Bookmark not defined.**

Supplementary Figure 5. SHAP partial dependence plots of religion and key predictors........... 8

Supplementary Method 1. Study tools... 9

Supplementary Figure 6. Calibration curves.. 10

Supplementary Table 1. Potential features and their level of missingness

Feature	Categories	Feature type	N (%) missing
**Sociodemographic features**			
Age in years		Numerical	0 (0)
Maternal religion	Christian<br>Islam<br>Traditional<br>Other (e.g. Atheists)	Categorical	0 (0)
Currently enrolled in any other program/project	Yes<br>No	Categorical	7 (0.35)
Marital status	Married<br>Unmarried	Categorical (derived)	0 (0)
Number of children between 1 to 5 years		Numerical	1 (0.05)
Number of pregnancies		Numerical	0 (0)
Number of children		Numerical	1 (0.05)
Child survival in all your pregnancies	Yes<br>No	Categorical	0 (0)
Caring for children with disability	Yes<br>No	Categorical	0 (0)
Live with partner	Yes<br>No	Categorical	225 (11.28)
**Socioeconomic status feature**			
Wealth index		Numerical (derived)	0 (0)
Education status	None (No formal education)<br>Formal education	Categorical (derived)	0 (0)
Partner's education status	None (No formal education)<br>Formal education	Categorical	0 (0)
**Health history features**			
Pregnancy complications	Yes<br>No	Categorical	8 (0.40)
Delivery problems	Yes<br>No	Categorical	8 (0.40)
Attended antenatal care	Yes<br>No	Categorical	8 (0.40)
Place of delivery	Hospital/clinic<br>Home	Categorical	0 (0)
Delivery assistant	Skilled (Doctor/nurse)<br>Unskilled (Relative, Traditional Birth Attendant (TBA), unassisted)	Categorical	8 (0.40)
Had health card	Yes<br>No	Categorical	0 (0)
**Food insecurity**			
Household food insecurity		Numerical (derived)	0 (0)
**Nutrition**			
Maternal BMI		Numerical (derived)	0 (0)

Supplementary Table 1 Legend: BMI - Body Mass Index, TBA - Traditional Birth Attendant

Supplementary Table 2. Features retained after one hot encoding

1	Currently enrolled in other program or project_Yes	
2	Marital status_Unmarried	
3	Live with partner_No	
4	Education status_None	
5	Religion_Christian	
6	Religion_Islam	
7	Religion_Traditional	
8	Child survival in all pregnancies_No	
9	Caring for children with disability_Yes	
10	Partner education status_None	
11	Had pregnancy complications_Yes	
12	Attended antenatal clinic_Yes	
13	Place of delivery_Hospital/clinic	
14	Had delivery problems_Yes	
15	Delivery assistant_Unskilled	
16	Had health clinic card_Yes	
17	Number of children between 1 to 5 years	
18	Number of children	
19	Number of times being pregnant	
20	Age	
21	Maternal BMI	
22	Household food insecurity scores	
23	Wealth index	

Supplementary Table 2 Legend: BMI - Body Mass Index

Supplementary Table 3. Hyperparameter tuning, optimal values for each model and random seed

Model	Hyperparameters	Explored values	Best value
Depressive symptoms prediction			
LR	C	0.0001, 0.001, 0.01, 0.1	0.01
RF	n_estimators	100, 200,300	200
	max_depth	1,3,5	3
	min_samples_leaf	22,24, 26	24
	max_features	14,16,18	14
XGBoost	n_estimators	1000, 2000, 3000	1000
	max_depth	1, 3, 5	1
	learning_rate	0.001,0.01,0.1	0.01
	min_child_weight	0.3	0.3
	colsample_bytree	0.3	0.3
Anxiety symptoms prediction			
LR	C	0.0001, 0.001, 0.01, 0.1	0.01
RF	n_estimators	100, 200, 300	100
	max_depth	1,2,5	2
	min_samples_leaf	28,30,32	28
	max_features	7,9,11	9,
XGBoost	n_estimators	3000, 4000,5000	3000
	max_depth	1,3,5	1
	learning_rate	0.0005,0.001,0.01,0.1	0.01

	min_child_weight	0.1	0.1
	colsample_bytree	0.1	0.1
A random seed (42) was used for data splitting and k-fold cross-validation to ensure reproducibility.			

Supplementary Table 3 Legend: LR - Logistic Regression; RF - Random Forest; XGBoost - Extreme Gradient Boosting; C - Inverse of regularization strength

Supplementary Figure 1. Correlation matrix of numerical features

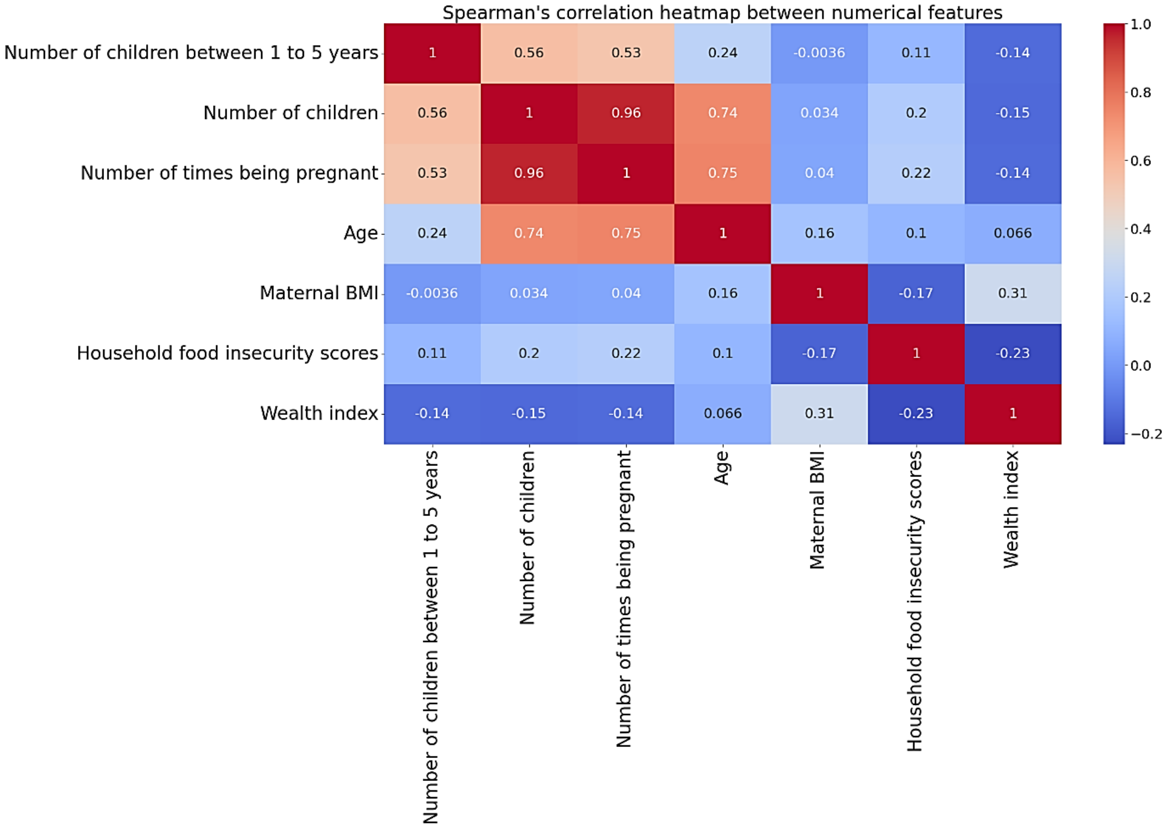

Supplementary Figure 1 Legend: BMI - Body Mass Index

Supplementary Figure 2. Correlation matrix of categorical features

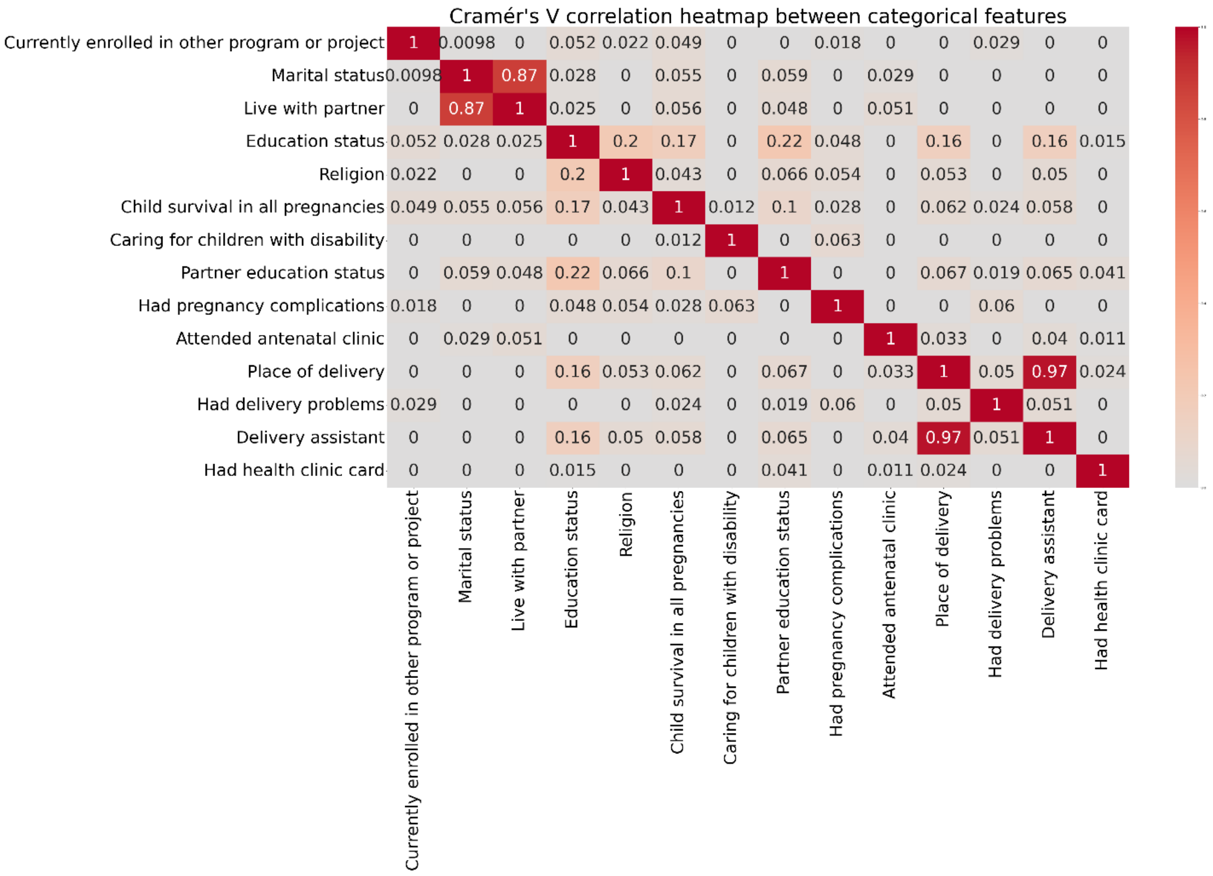

Supplementary Figure 3. SHAP impact and partial dependence plots for predicting depressive symptoms by Random Forest

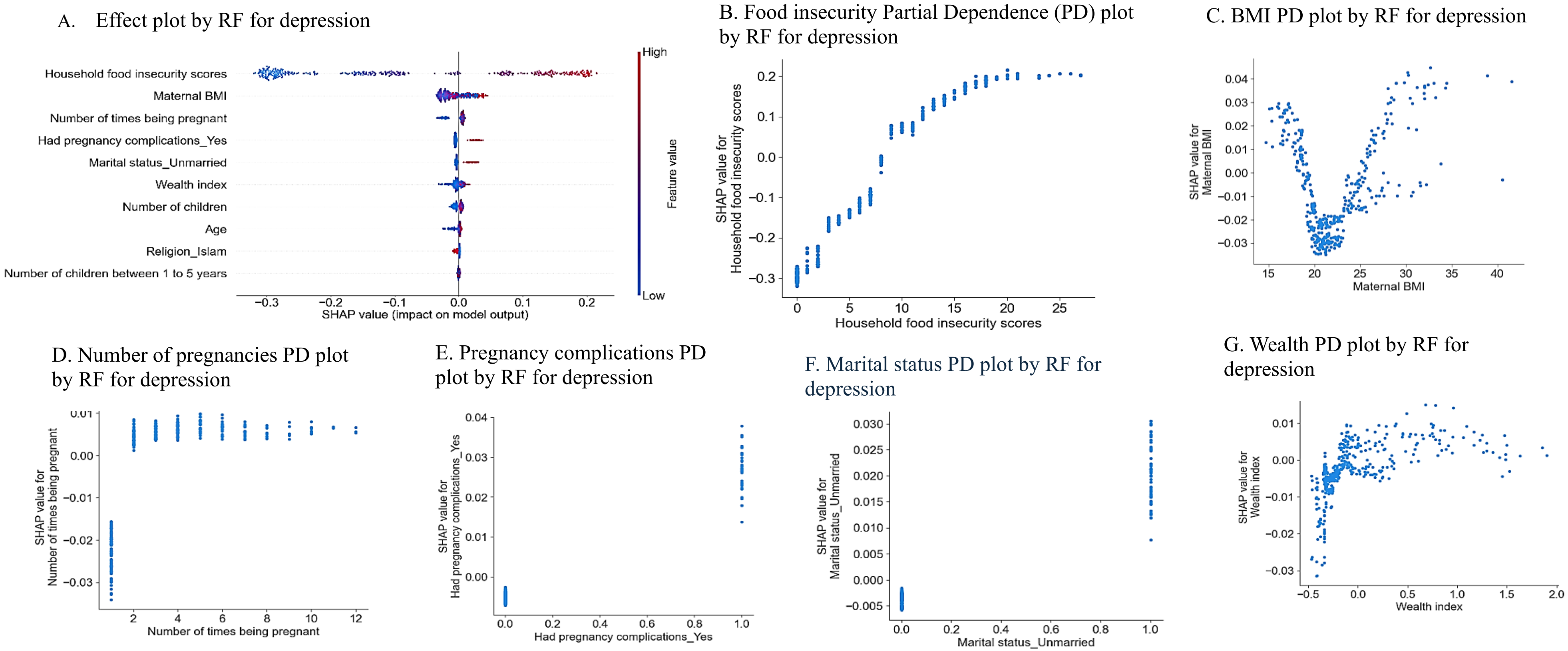

Supplementary Figure 3 Legend: Plot A: A SHAP beeswarm plot showing the top 10 important features influencing depressive symptoms from a Random Forest (RF) model, ranked by importance. Color indicates the feature value (red = high, blue = low), and position along the x-axis shows the direction and strength of impact. Plots B–G present SHAP partial dependence for the top 6 key predictors: increased food insecurity, number of pregnancies, as well as low BMI, low wealth index, pregnancy complications and unmarried marital status, all of which have a positive impact on prediction of depressive symptoms.

Supplementary Figure 4. SHAP impact and partial dependence plots for predicting anxiety symptoms by Random Forest

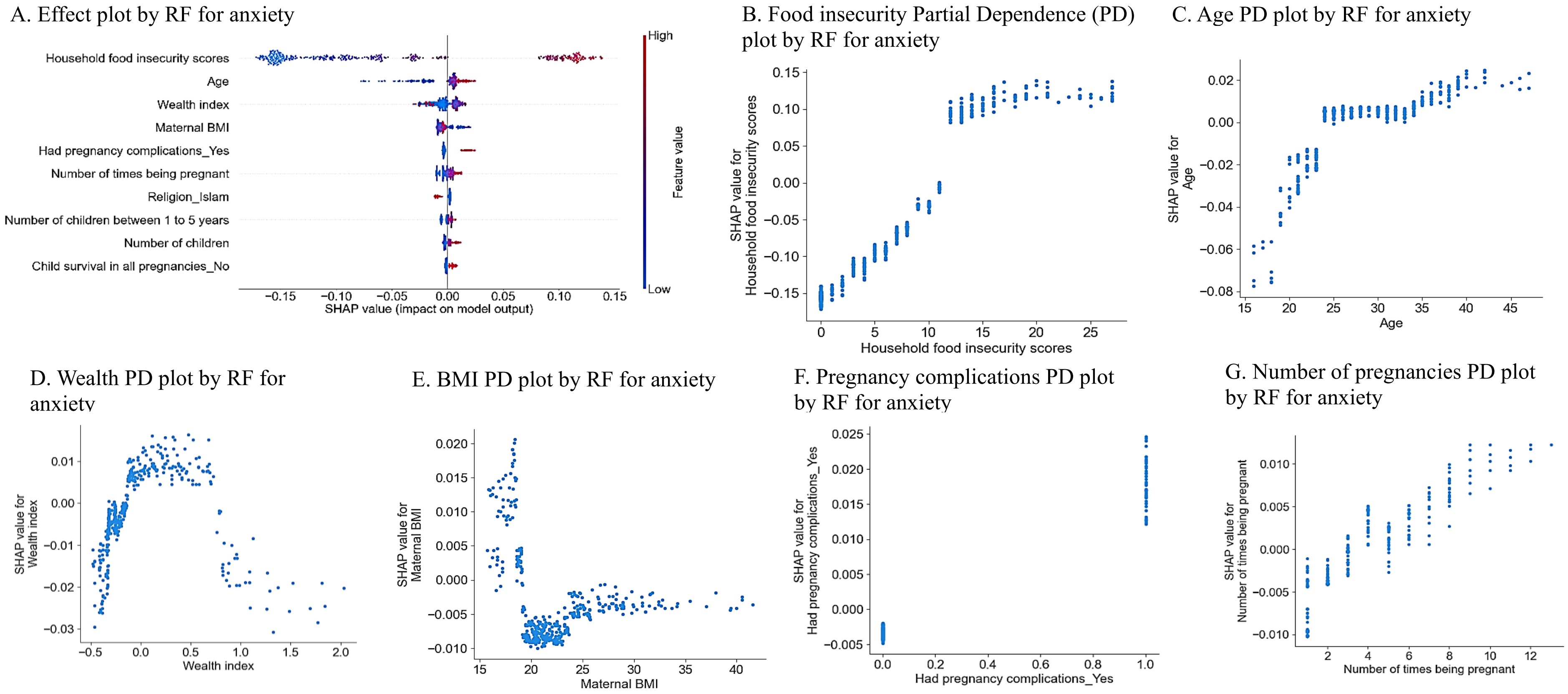

Supplementary Figure 4 Legend: Plot A: A SHAP beeswarm plot showing the top 10 important features influencing anxiety symptoms from a Random Forest (RF) model, ranked by importance. Color indicates the feature value (red = high, blue = low), and position along the x-axis shows the direction and strength of impact. Plots B–G present SHAP partial dependence for the top 6 key predictors: increased food insecurity, age, and number of pregnancies, as well as low wealth index, low BMI, and pregnancy complications, all of which have a positive impact on prediction of anxiety symptoms.

Supplementary Figure 5. SHAP partial dependence plots of religion and key predictors

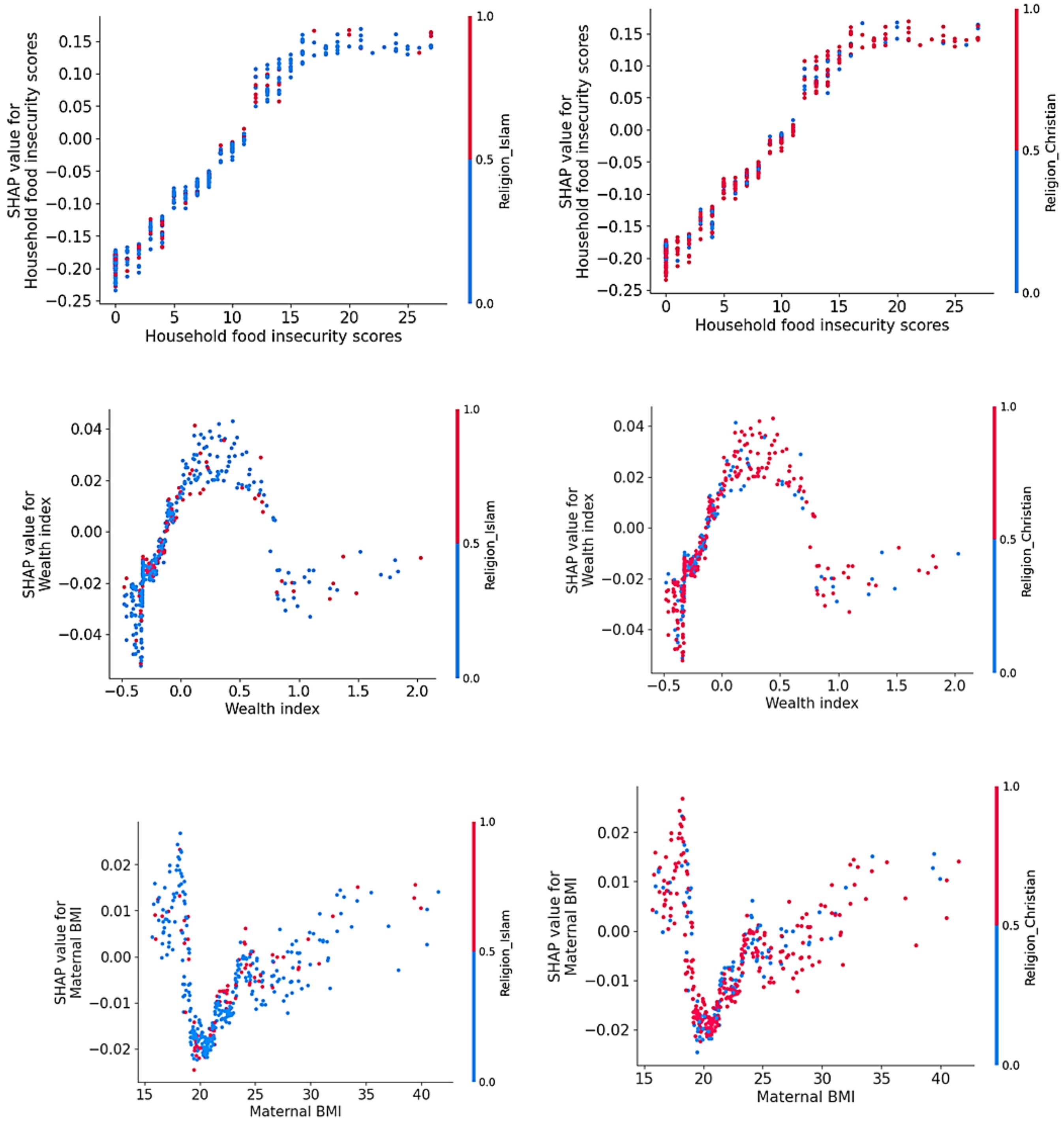

Supplementary Figure 5 Legend: SHAP partial dependence plots illustrating the interactions between religion and key predictors; food insecurity, wealth index, and maternal BMI.

Supplementary Method 1. Study tools

Household Food Insecurity Access Scale (HFIAS)

The HFIAS questionnaire [1,2] was used to assess household food insecurity levels. It includes 9 items evaluating aspects such as food supply uncertainty, insufficiency in the quality and quantity of food, going to bed hungry, and enduring full days and nights without eating over the past four weeks. The total score, calculated by summing the individual item scores, ranges from 0 to 27, with higher scores indicating greater levels of food insecurity. The HFIAS has been validated through various studies in developing countries.[3-6]

Social demographic and economic questionnaire

The sociodemographic data collected for this study included age, marital status, religion, and other additional characteristics. Socioeconomic status was assessed using a 10-asset items commonly applied in the Kenyan context, which covers ownership of assets such as a radio, television, video machine, fridge/freezer, cooker, computer/tablet, bicycle, motorcycle, car/truck, and telephone.[7] Each owned asset is assigned a value of 1, while the absence of an asset is scored as 0. In addition to assessing socioeconomic status, housing quality was evaluated based on specific characteristics, including the primary materials used for constructing floors (e.g., tiles, mud), walls (e.g., mud, bricks), and roofs (e.g., iron sheets, grass). Other factors considered included the type of toilet facilities (e.g., flush, bush) and the cooking area location (e.g., kitchen, outside the house), among others. These features were subsequently categorized into two classes: "improved" assigned a value of 1 or "unimproved" assigned a value of 0 [8,9]). A single wealth index score was calculated using the Multiple Correspondence Analysis (MCA) technique, which is particularly suited for binary data.[10]

Clinical and health history questionnaire

A questionnaire with yes/no items was used to collect health history information for the mothers. The questionnaire addressed pregnancy-related complications, delivery challenges, and other relevant health factors.

Anthropometric measurements

Height and weight measurements were taken to calculate Body Mass Index (BMI), which was used as an indicator of maternal nutritional status. Measurements followed WHO-recommended procedures,[11] and for quality control, each was taken three times.

Supplementary Figure 6. Calibration curves

A. Calibration curves for depressive symptoms prediction

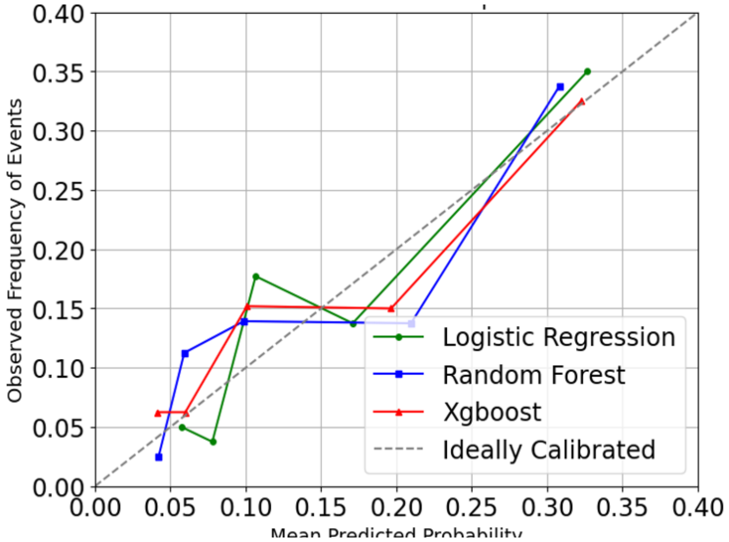

B. Calibration curves for anxiety symptoms prediction

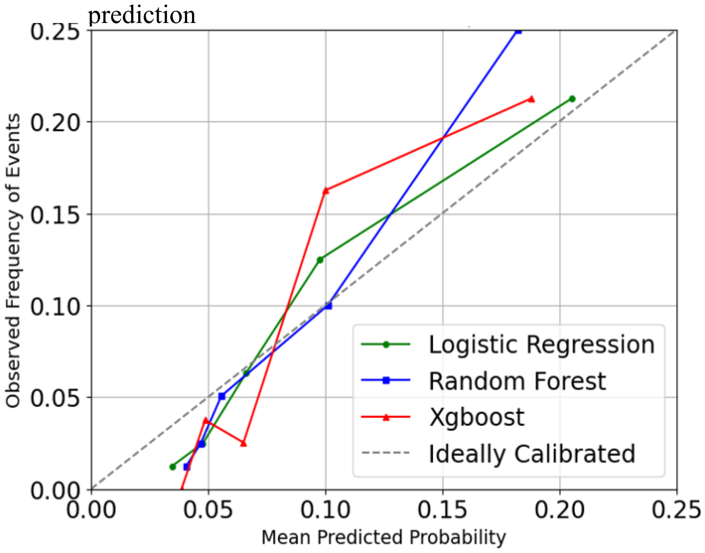

Supplementary Figure 6 Legend: Calibration plots assessing model accuracy. A Calibration curve for depressive symptoms prediction on test set. Brier scores were 0.117, 0.119, and 0.118 for Ridge Logistic Regression, Random Forest, and XGBoost, respectively. The Hosmer–Lemeshow goodness-of-fit test indicated no evidence of poor calibration for any model (P = 0.203 for Ridge Logistic Regression, P = 0.254 for Random Forest, and P = 0.595 for XGBoost). B Calibration curve for anxiety symptoms prediction on test set. Brier scores were identical across models (0.074 for Ridge Logistic Regression, Random Forest, and XGBoost). The Hosmer–Lemeshow goodness-of-fit test showed no evidence of poor calibration (P = 0.069 for Logistic Regression, P = 0.494 for Random Forest, and P = 0.066 for XGBoost).

References

1. Swindale A, Bilinsky P. Development of a universally applicable household food insecurity measurement tool: process, current status, and outstanding issues. *The Journal of nutrition.* 2006;136(5):1449S-1452S.
2. Coates J, Swindale A, Bilinsky P. Household Food Insecurity Access Scale (HFIAS) for measurement of food access: indicator guide: version 3. 2007.
3. Maes KC, Hadley C, Tesfaye F, Shifferaw S, Tesfaye YA. Food insecurity among volunteer AIDS caregivers in Addis Ababa, Ethiopia was highly prevalent but buffered from the 2008 food crisis. *The Journal of nutrition.* 2009;139(9):1758-1764.
4. Becquey E, Martin-Prevel Y, Traissac P, Dembélé B, Bambara A, Delpeuch F. The household food insecurity access scale and an index-member dietary diversity score contribute valid and complementary information on household food insecurity in an urban West-African setting. *The Journal of nutrition.* 2010;140(12):2233-2240.
5. Knueppel D, Demment M, Kaiser L. Validation of the household food insecurity access scale in rural Tanzania. *Public health nutrition.* 2010;13(3):360-367.
6. Mohammadi F, Omidvar N, Houshiar-Rad A, Khoshfetrat M-R, Abdollahi M, Mehrabi Y. Validity of an adapted Household Food Insecurity Access Scale in urban households in Iran. *Public health nutrition.* 2012;15(1):149-157.
7. Abubakar A, Van de Vijver F, Van Baar A, et al. Socioeconomic status, anthropometric status, and psychomotor development of Kenyan children from resource-limited settings: a path-analytic study. *Early human development.* 2008;84(9):613-621.
8. Habitat U. Module 1: adequate housing and slum upgrading. *SDG indicator.* 2018;11(1).
9. Florey L, Taylor C. Using household survey data to explore the effects of improved housing conditions on malaria infection in children in sub-Saharan Africa. 2016.
10. Booysen F, Van Der Berg S, Burger R, Von Maltitz M, Du Rand G. Using an asset index to assess trends in poverty in seven Sub-Saharan African countries. *World Development.* 2008;36(6):1113-1130.
11. Status WP. The use and interpretation of anthropometry. *WHO technical report series.* 1995;854(9).
